# Synthesis and Catalytic Study of NiAg Bimetallic Core–Shell Nanoparticles

**DOI:** 10.3390/ma16020659

**Published:** 2023-01-10

**Authors:** Konrad Wojtaszek, Filip Cebula, Bogdan Rutkowski, Magdalena Wytrwal, Edit Csapó, Marek Wojnicki

**Affiliations:** 1 Faculty of Non–Ferrous Metals, AGH University of Science and Technology, al. A. Mickiewicza 30, 30-059 Krakow, Poland; 2 Faculty of Metals Engineering and Industrial Computer Science, AGH University of Science and Technology, al. A. Mickiewicza 30, 30-059 Krakow, Poland; 3 Academic Centre for Materials and Nanotechnology, AGH University of Science and Technology, al. A. Mickiewicza 30, 30-059 Krakow, Poland; 4 MTA-SZTE “Lendület” Momentum Noble Metal Nanostructures Research Group, University of Szeged, Rerrich B. sqr. 1, H-6720 Szeged, Hungary

**Keywords:** bimetallic nanoparticles, core–shell nanoparticles, catalytic properties, methylene blue reduction, kinetic studies

## Abstract

This publication presents the synthesis of core–shell nanoparticles, where the core was Ni, and the shell was a Ag–Ni nano alloy. The synthesis was based on the reduction of Ni and Ag ions with sodium borohydride in the presence of trisodium citrate as a stabilizer. In order to determine the phase composition of the obtained nanoparticles, an XRD study was performed, and in order to identify the oxidation states of the nanoparticle components, an XPS spectroscopic study was performed. The composition and shape of the particles were determined using the HR-TEM EDS test. The obtained nanoparticles had a size of 11 nm. The research on catalytic properties was carried out in the model methylene blue reduction system. The investigation of the catalytic activity of colloids was carried out with the use of UV–Vis spectrophotometry. The Ag–Ni alloy was about ten times more active than were pure silver nanoparticles of a similar size.

## 1. Introduction

The constant, high production of textiles, leather, plastic, cosmetics, paper, and pharmaceuticals, as well as food processing, printing, and dye manufacturing, cause a significant production of wastewater rich in organic dyes and pigments [1,2]. 

Dyes can be categorized into the following classes: anthraquinone, azo, reactive, disperse, acidic, basic, and neutral dyes. Anthraquinone and azo dyes are the most popular and are commonly used in industry. They account for more than 60% of reactive dyes and are extensively used in the textile industry [3,4,5]. As much as 20% of dye residues are released into industrial effluents and disposed into receiving waters without effective purification or treatment. These dyes have been declared carcinogenic and tumorigenic by the International Agency for Research on Cancer (IARC) and the National Institute for Occupational Safety and Health; however, these dyes are still used in textile dyeing processes [6]. Organic dyes are complex due to their many aromatic rings and their synthetic origin. These dyes are highly stable, resistant to photo and biological degradation, and refractory against chemical oxidation, and their discharge into the environment poses a severe threat to the sustainability of ecosystems. Over time, water contamination by organic dyes has become a real threat to aquatic ecosystems [7,8,9]. Even a small concentration of these dyes can create intense color, which absorbs a significant amount of sunlight, effectively interfering with photosynthesis in aquatic life. The consequence of this pollution is a substantial disturbance of the ecological balance in the ecosystem [10,11]. 

Despite these disadvantages, due to their low cost and easy mixing, it is difficult to replace these materials on an industrial scale. Many dye removal methods are currently being implemented, including physical and chemical methods [12,13,14]. Removing the dyes is still challenging due to the high cost and the production of sludge, which requires further processing. Therefore, the development of new, low-cost methods is required.

Over the years, the application of metal nanoparticles has achieved tremendous progress in academic and industrial sectors [15]. Due to the high surface-to-volume ratio, nanostructures exhibit explicit and extraordinary properties. They differ from bulk materials in their unusual packing of atoms in crystals, which is only possible on a nanoscale. The unique optical [16], photonic [17], electrochemical [18], magnetic [19], electrical, and catalytic properties of metal nanoparticles make them an attractive choice for researchers and manufacturers. There are widespread chemical and physical pathways for producing metal nanoparticles with controllable shape, size, and surface properties [20]. Although it has incredible potential and numerous applications, the synthesis of nanoparticles still comes with many challenges. The difficulties regarding reproducibility, along with the often high production costs, drive steady interest in synthesizing nanoparticles with controlled size and shape using inexpensive and environmentally friendly materials.

It is already well established that noble metals are suitable catalysts for degrading dyes [21,22,23,24]. Despite this, there are two main problems with these materials. The high price of products and materials is one of the limiting elements in the water purification market. An optimal way to recover the catalyst after the process is also a critical problem. Researchers have focused on synthesizing bimetallic nanoparticles, such as AgNiNPs, to address these problems. Adding a magnetic element allows an external magnetic field to collect catalysts after the process and reduces the amount of silver in the material, thus reducing its cost.

The synthesis of silver nanoparticles is a well-known process. Different reducing agents [25], as well as reactor types [26], can be used, and the catalytic properties of AgNPs have been widely investigated [27,28]. The synthesis of silver-based nano alloys opens up completely new possibilities. First of all, it is possible to obtain alloys that are not obtainable in the macro world [29].

The aim of this work was to investigate the possibility of synthesis, along with the catalytic properties, of AgNi nanoparticles. For this purpose, different methods, including laser ablation [30], reduction by green plant extracts [31,32], chemical reduction by NaBH_4_ [33,34], and solvothermal processes [35,36], may be applied. There are many works devoted to the synthesis of AgNi nanoparticles. Most of them focus on optical [37], biological [38], and magnetic [39] properties. Only a few of them are focused on the catalytic properties in the reduction reaction of organic dyes.

Therefore, in this paper, we have studied the synthesis of core–shell AgNi nanoparticles for the catalytic reduction of organic dyes. The reduction of the methylene blue process was selected as a reference reaction. 

## 2. Experimental Section

### 2.1. Materials

Analytically pure formulations of silver nitrate (AgNO_3_), silver sulfate (Ag_2_SO_4_), trisodium citrate dihydrate (TSC), methylene blue (MB), and sodium borohydride (NaBH_4_) were obtained from POCH (Poland). Analytically pure nickel sulfate hexahydrate (NiSO_4_·6H_2_O) was obtained from Acros Organics B.V.B.A. Deionized water was used in all experiments and was supplied from deionizer Polwater DL3N-150 (Kraków, Poland).

### 2.2. Synthesis of Nanomaterials

#### 2.2.1. Synthesis of Ag Nanoparticles

Silver nanoparticles were prepared by reducing AgNO_3_ with NaBH_4_ in an ambient atmosphere and at room temperature. First, an appropriate amount of AgNO_3_ salt was dissolved in 25 mL of deionized water to obtain a 0.5 mM solution. A similar procedure was performed to obtain 0.5 mM trisodium citrate solution. A total of 25 mL of 0.5 mM AgNO_3_ was mixed with 25 mL of 0.5 mM trisodium citrate in a small beaker (100 mL) using a magnetic stirrer. Next, an appropriate amount of NaBH_4_ was dissolved in 25 mL of deionized water to obtain a 10 mM solution. After that, 1.5 mL of freshly prepared NaBH_4_ solution was added to the solution containing Ag^+^ ions. After ca. 5 min, the reaction stopped, and the final color (light yellow) appeared.

#### 2.2.2. Synthesis of AgNi Nanoparticles

Fine AgNi bimetallic nanoparticles were prepared by reducing NiSO_4_ hydrate with sodium borohydride in an ambient atmosphere and at room temperature. An total of 0.25 mL of 10 mM NiSO_4_ hexahydrate and 0.25 mL of 10 mM trisodium citrate were added to 19 mL of deionized water and mixed. After 15 min of stabilization, 0.5 mL of 10 mM freshly prepared NaBH_4_ was added and rapidly mixed for 2 min. The solution was left undisturbed for 1 h, and after approximately 40 min, the solution started changing color to dark brown/black. Next, 0.125 mL of Ag_2_SO_4_ was added to the solution and rapidly mixed for 1 min. The final color of the solution was dark yellow/light brown.

### 2.3. Characterization of Obtained Nanomaterials

A Jasco V-770 spectrophotometer (Tokyo, Japan) was used to study the UV–Vis spectra of the Ag and AgNi nanoparticles. The crystal structures of the obtained colloids were analyzed using a Rigaku Miniflex II X-ray diffractometer (XRD) with Cu-Kα (40 kV, 40 mA, k = 1.5406 Å) radiation and scanning angles between 10° and 90°. For this purpose, colloid was evaporated on the glass substrate to obtain the appropriate amount of dry mass. Size and zeta potential were measured using a Malvern Zetasizer Nano-ZS (Worcestershire, UK). Measurements were performed at 20 °C. The morphology and elemental distribution were determined using an HR-STEM Titan^3^ G2 60–300 (300 kV, probe Cs corrected) equipped with the ChemiSTEM system (Thermo Fisher Scientific, Eindhoven, The Netherlands). Solutions of the obtained nanoparticles were dropped on copper mesh covered with amorphous carbon film (thickness ca. 30 nm), and then placed onto a sample holder. Using the ImageJ application (ImageJ software version 1.52V, Wayne Rasband, National Institutes of Health, USA), the size of the particles was measured from HR-STEM pictures.

The scanning X-ray photoelectron spectrometer PHI 5000 Versa Probe II (ULVAC-PHI, Chigasaki, Japan) system, with a microfocused (100 µm, 25 W) Al Kα X-ray beam, was used to perform an elemental analysis of the samples. The photoelectron spectra were acquired from 400 µm × 400 µm areas with an analyzer pass energy of 46.95 eV and a photoelectron take-off angle of 45°. The charging effect was compensated for using a dual-beam charge neutralizer. The operating pressure in the analytical chamber was <5 × 10^−7^ Pa. Measurements were performed at two independent areas for each sample. All XPS peaks were referenced to the C1s C-C line at 285.0 eV. Spectra were deconvoluted using PHI MultiPak software (version 9.9.2), and the spectrum background was subtracted using the Shirley method.

### 2.4. Catalytic Tests 

A stock solution of methylene blue (MB) was prepared by the dissolution of 1 g of methylene blue in 1 L of deionized water. Then, an adequate amount of the solution was diluted to obtain 2.7 mL of 0.1 M MB solution. Next, this volume was heated up to the temperature of the given test. After that, 100 µL of catalyst was added to the dye solution, and finally, 0.2 mL of 0.1 M NaBH_4_ was added to the cuvette. The final volume of the reacting solutions was 3 mL. The absorbance of the dye was measured using a Jasco V-770 spectrometer (Jasco, Tokyo, Japan). Tests were performed under continuous mixing (500 rpm) using a built-in magnetic stirrer. Absorbance was measured between 220 to 750 nm, with 2 min intervals between each measurement. The tests were performed for 3 systems: without a catalyst, with Ag nanoparticles as catalysts, and with Ag–Ni alloy as a catalyst. For each system, experiments were performed at five different temperatures, 25, 30, 35, 40, and 45 °C. All experiments were repeated a minimum of 3 times.

## 3. Results and Discussion

### 3.1. Ag Nanoparticles Synthesis

For the synthesis of silver nanoparticles (AgNPs), the well-known method of reduction by sodium borohydride was used. This method employs trisodium citrate as a capping and stabilizing agent. A few minutes after adding the reducing agent, the solution changed color to yellow, indicating the formation of AgNPs.

### 3.2. AgNi Bimetallic Nanoparticles Synthesis

To obtain AgNi bimetallic nanoparticles, a two-step route was chosen. In the first step, nickel nanoparticles (NiNPs) were produced. As with the silver nanoparticles, trisodium citrate was used as a stabilizer and capping agent. This reduction reaction is highly susceptible to the reverse reaction of dissolved NiNPs caused by dissolved oxygen. By controlling the accessibility of oxygen to the solution, we adjusted the reaction speed and the resulting size of the obtained nanoparticles. The downside of this approach is the surface oxidation of the nanoparticles; after the reducer’s degradation, the process of dissolution of nanoparticles starts. As Aparna Roy et al. reported [40], the reduction of nickel ions by NaBH_4_ in the presence of oxygen can result in the formation of tetragonal NiNPs, which do not show magnetic properties at room temperature. In our case, the small size, lack of magnetic properties, and similar synthesis conditions allowed us to assume that tetragonal NiNPs were synthesized.

In the second step, an appropriate amount of silver sulfate was added to the prepared Ni colloid solution. Immediately, the colloid solution changed color to dark yellow/light brown, which confirmed that the silver created a shell structure on the surface of the NiNPs. The silver coating was accomplished via the following reaction:

(1) $$Ni^{0}+2Ag^{+}\to \;2Ag^{0}+Ni^{2+}$$

which occurred at the surface of the Ni particles. Due to this reaction, the surface of Ni should have been partially replaced by a uniform layer of Ag. Usually, the metal coating takes on the microstructure of the substrate’s microstructure. This is the so-called templating effect. It was expected that the silver would crystalize into a tetragonal structure. 

### 3.3. Characterization of Obtained Colloids

#### 3.3.1. Indication of the Presence of Nanoparticles

**Figure 1:**
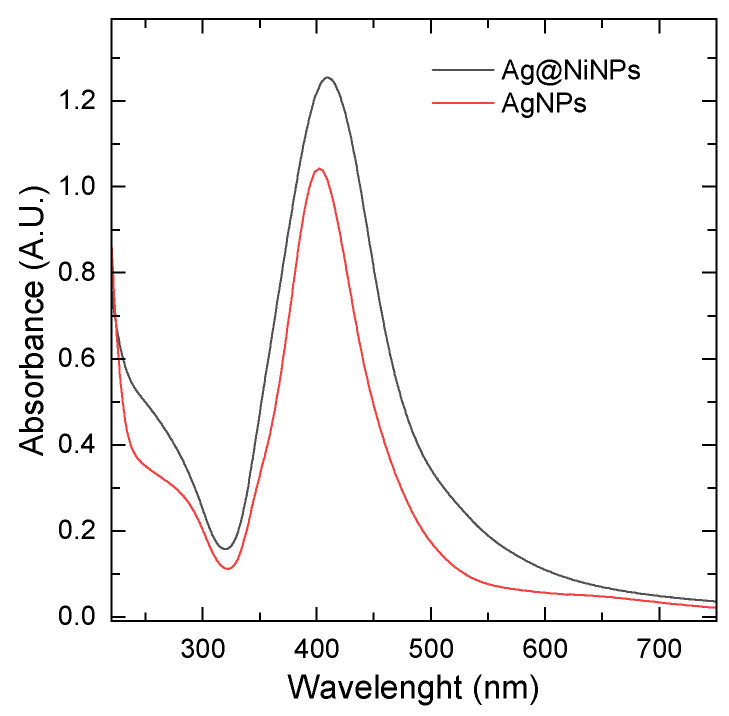
UV–VIS spectra of obtained AgNPs and Ag@NiNPs.

UV–Vis spectroscopy was the first method used to confirm the synthesis of AgNPs and Ag coating on NiNPs (Ag@NiNPs). In Figure 1, the UV–Vis spectrum for the suspension of AgNPs and Ag@NiNPs can be seen. The maximum absorbance for AgNPs was at 402 nm, and for Ag@NiNPs, it was at 409 nm, which confirms the presence of silver nanoparticles in both mixtures. A sharp peak indicates the presence of spherical or round-shaped nanoparticles with a narrow size distribution. 

Pure nickel nanoparticles did not show any peaks, but only a background shift due to light scattering. Ag@NiNPs exhibited higher absorbance compared to AgNPs. The peak was slightly shifted to longer wavelengths (red shift). 

This suggests that the radius of Ag@NiNPs was greater than that of pure Ag, which is understandable, as the assumption is that Ag should form a layer on the Ni surface. Thus, the hydrodynamic radius of Ag@NiNPs should be greater than that of pure AgNPs.

A. A. Akinsiku et al. noted that the presence of nickel in the nanohybrid resulted in a blue shift in the absorbance wavelengths when compared with the corresponding monometallic AgNPs (341 to 327 nm) [31]. This observation is opposite to ours, where a red shift was observed. However, it should be noted that A. A. Aknisiku used green plant extract for the nanohybrid synthesis, and the organic chemical compounds can effectively adsorb at the surface of Ag and significantly change the position of surface plasmon resonance.

#### 3.3.2. XRD Analysis

**Figure 2:**
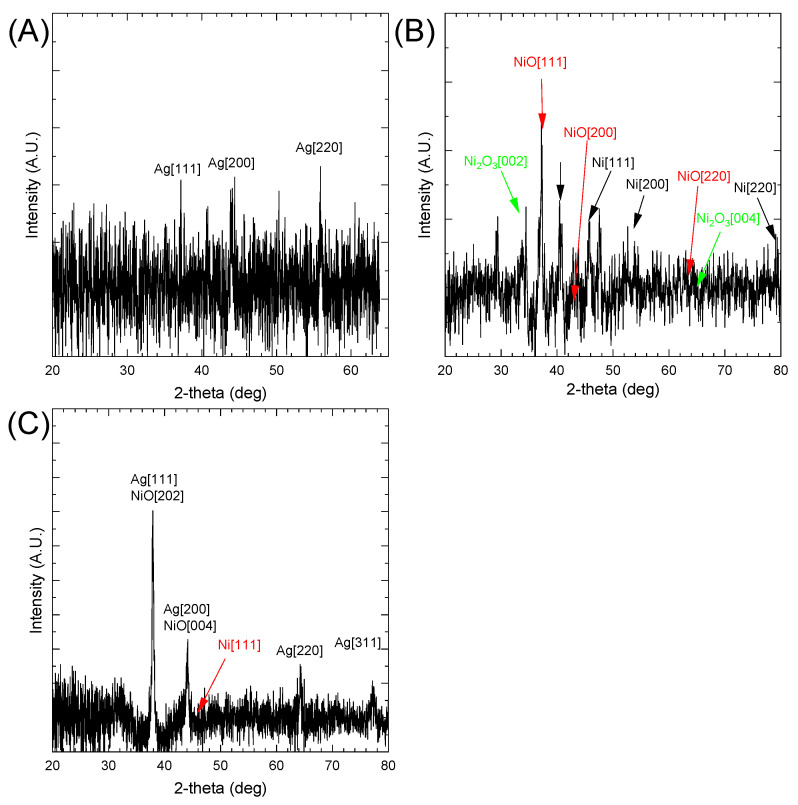
XRD patterns of (**A**) AgNPs, (**B**) NiNPs, and (**C**) Ag@NiNPs.

X-ray diffraction analysis is an analytical technique for investigating the crystalline structure of AgNPs and Ag@NiNPs. The XRD spectrum for both samples is presented in Figure 2. The spectrum obtained for Ag@NiNPs (Figure 2A) shows visible peaks from silver and overlapping peaks from Ni_15_O_16_, which have a tetragonal crystal structure. This phase confirmed the successful synthesis of tetragonal nickel nanoparticles stabilized by oxygen, although silver could be spotted in the FCC structure. The reason behind this may be that the internal stress of the lattice was too high for a tetragonal structure, inevitably leading to the transformation into an FCC structure. In Figure 2B, the XRD spectrum for the AgNPs sample is presented. The peaks were harder to observe. This suggests that the AgNPs were smaller than the Ag@NiNPs. However, the peaks around 38° and 44°, which can be attributed to silver, were easily detectable. The third distinguishable peak has been attributed to silver oxide. However, we do not have strong confirmation of the formation of silver oxide. 

The XRD analysis of Ag, Ni, and AgNi nanoparticles was performed. The obtained results are shown in Figure 2A–C, respectively. The database suggests the presence of the following compounds in the tested sample: (A) FCC Ag, (B) cubic NiO (Card No.: 01-073-1519), cubic Ni (Card No.: 01-088-2326), and Ni_2_O_3_ [41]. Due to the nano size, the peaks are strongly broadened. Therefore, it cannot be excluded that NiOx was also present in the sample (where x -0.81–097; Card No.: 01-078-4376, Card No.: 01-078-4372, Card No.: 01-078-4380). Non-stoichiometric compounds shifted the reflex position [101] from 36.82 to 37.22 deg. The formation of non-stoichiometric oxides can be directly related to sample preparation for XRD analysis. In the first stage, it is required to dry the sample in a protective gas atmosphere. Unfortunately, the analysis itself is carried out without a protective atmosphere, which can lead to secondary oxidation of the sample and formation of non-stoichiometric compounds. The database does not contain information on the existence of the Ag–Ni alloy. 

Using the Scherrer equation (see Equation (2)), it is possible to calculate crystal size.

(2) $$d=\frac{K\cdot \lambda }{\beta \cdot \cos\Theta }$$

where *K* is the dimensionless shape factor (here: 0.95), *λ* is the wavelength of Cu_Kα_ radiation, *Q* is the Bragg angle, *β* is the line broadening at half of the peak maximum, and *d* is the calculated size of the single crystallite.

The peak located at 44° registered for the AgNPs sample was 10.6 nm. The mean value for all four registered peaks was 15.8 ± 11 nm. The crystallite size calculated for the (200) peak of AgNPs was 2.9 nm. For NiNPs (Ni [111] peak), the calculated grain size was 12.7 nm. In the case of Ag@NiNPs, the calculated NiNPs (Ni [111] peak) grain size was 10.1 nm. This suggests that the process of Ag deposition on the surface of NiNPs is associated with an additional reaction between metallic Ni and Ag^+^ ions. 

#### 3.3.3. Nanoparticle Size and Size Distribution 

**Figure 3:**
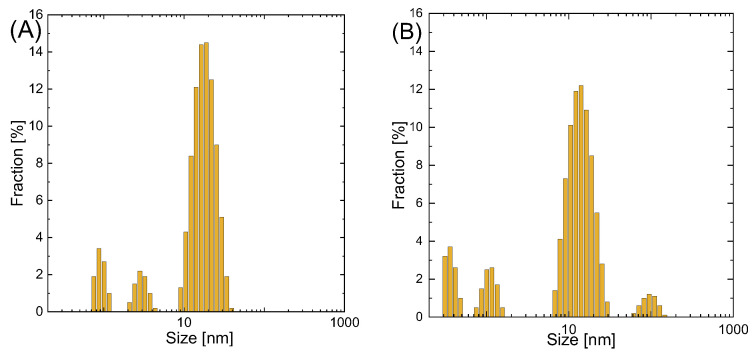
Size distribution histograms of (**A**) AgNPs and (**B**) Ag@NiNPs.

Nanoparticle size and size distribution are shown in Figure 3A,B. The average particle size was 18 nm for AgNPs and 14 nm for Ag@NiNPs. In the pictures, one can also see a minor volume of smaller particles (around 1 nm) and, in the case of Ag@NiNPs particles, around 100 nm. This shows that nickel nanoparticles tend to create agglomerates, even with the addition of trisodium citrate. 

The precursor undergoes a galvanic replacement reaction during the synthesis of core–shell nanoparticles. Metals ions, after the reduction reaction, create the shell. Thus, an increase in particle size is expected. This reaction results in a uniform layer, the shell, with no significant change in particle size. 

This suggests that the layer thickness was on the order of 1–2 nm. Additionally, it should be noted that in the case of AgNPs and Ag@NiNPs, two fractions with principal sizes 0.8 nm and 2.7 nm were observed. It is suggested that these two peaks were related to the formation of nanobubbles of H_2_ formed by decomposition of NaBH_4_ in the solution. The existence of nanobubbles in aquatic systems has already been described in the literature [42]. The fourth peak observed in Figure 3B was probably related to aggregates of Ag@NiNPs. Therefore, TEM analysis was required to confirm these assumptions.

#### 3.3.4. Morphology and Chemical Composition Study

**Figure 4:**
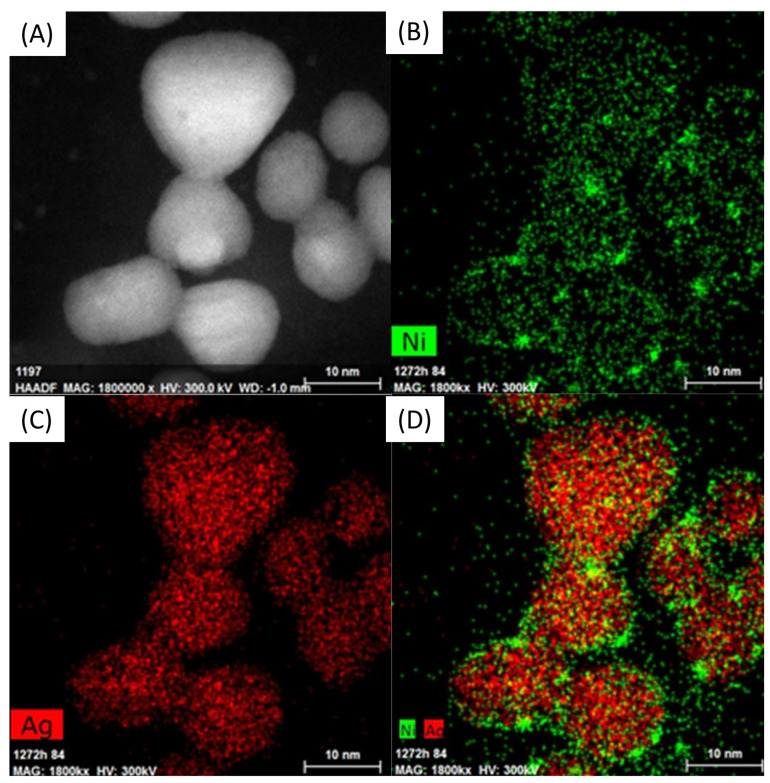
Distribution of elements in AgNiNPs, (**A**) HR-SEM image, (**B**) Ni distribution, (**C**) Ag distribution, and (**D**) Ni and Ag distribution.

**Figure 5:**
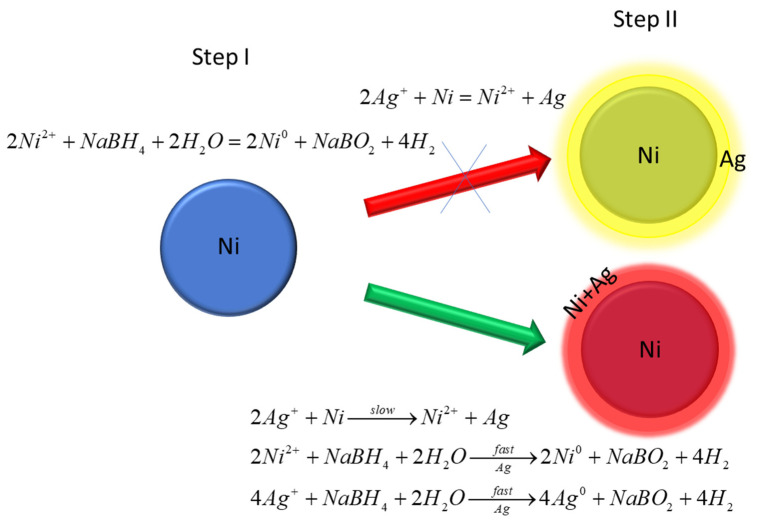
Diagram of the formation of the outer layer in the core–shell system.

Figure 4 shows the distribution of elements in AgNiNPs. The images indicate that the core–shell structure cannot be confirmed. Nickel atoms were highly dispersed and observed at the surface of the nanoparticles. This was caused by a chemical reaction, which resulted in the reduction of silver and the oxidation of nickel, causing the release of Ni^2+^ ions into the solution. Next, the excess or reducing agent also reduced the Ni^2+^ ions. The reaction mechanism is schematically shown in Figure 5.

The expected result of this reaction was the creation of a silver shell around the nickel core. Instead, an alloy nanoparticle was created, despite the total immiscibility of the components. One of the consequences of this phenomenon was a lower than anticipated nickel content in the alloy due to the dissolution of Ni atoms. The creation of alloy nanoparticles also explains the loss of the tetragonal structure. Due to differences in atom size, the lattice misfit was too large to maintain the current crystal structure. The stress caused by this misfit was the driving force behind the transformation into the FCC structure.

In Figure 4, the EDS elemental map is visible. It confirms that the core particle was based on silver, whereas its shell was enriched with nickel.

**Figure 6:**
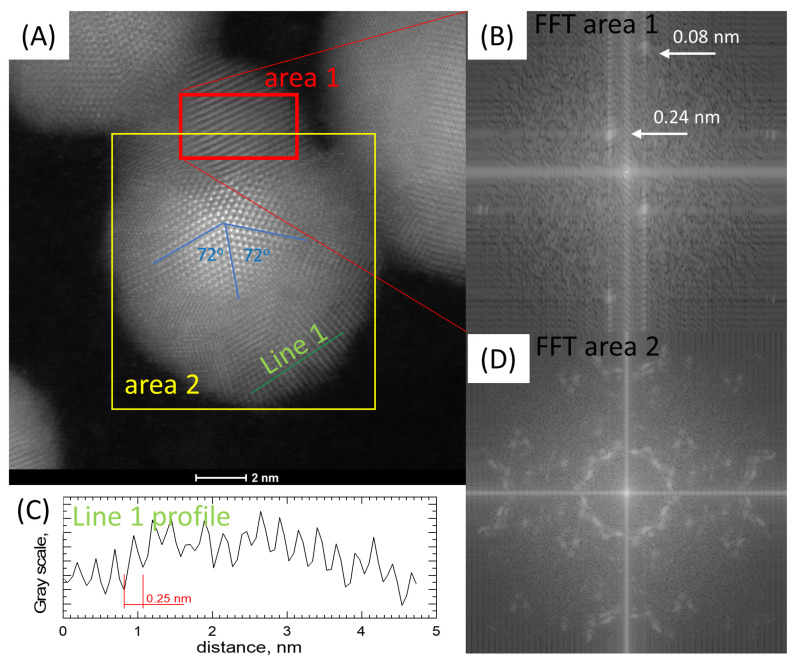
HR-TEM image and analysis. (**A**) fold symmetry of AgNiNPs, (**B**) FFT analysis of selected area 1, (**C**) line profile analysis of selected direction, and (**D**) FFT analysis of selected area 2.

In Figure 6, a round particle exhibiting five-fold symmetry can be seen. This symmetry is only allowed in small particles and quasi-crystals. Various multiply twinned particles with five-fold symmetry have been reported for face-centered cubic FCC structures in the early stages of particle growth. In Figure 6B,C, the FFT and line profile analyses are shown. These studies indicate that the distance between the atomic layers equals around 0.24 nm, corresponding to the distance between layers for the Ag [111] planes. The other pointed distance is extremely small and suggests a highly twinned structure, typical for five-fold symmetry particles. Similar observations were noted by K. Mukesh and D. Sasanka [43]. Figure 6D shows an FFT analysis of the area corresponding to most of the particles. The results show repeated triple dot patterns, which confirms the system symmetry. 

**Figure 7:**
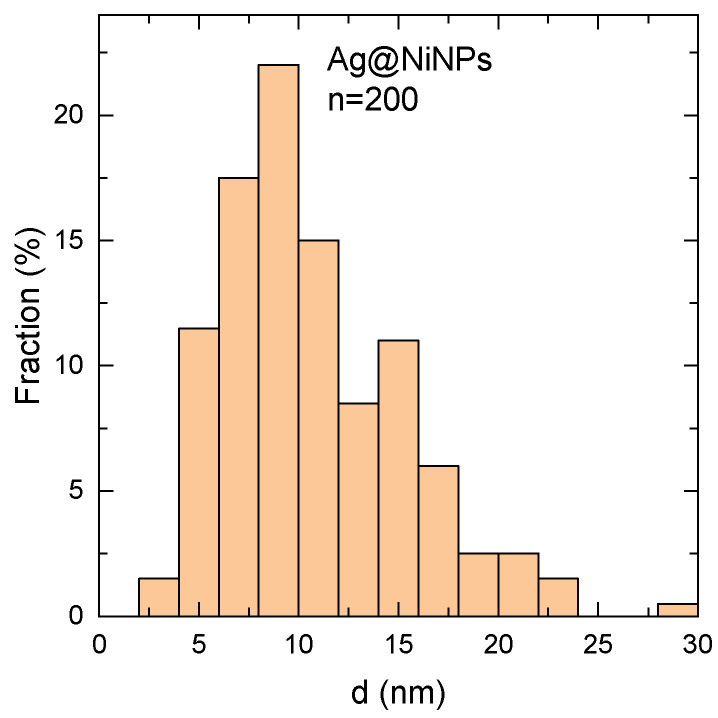
Size distribution based on HR-TEM pictures in which the number of analyzed particles is 200.

Figure 7 presents the size distribution of Ag@NiNPs measured from TEM images using the ImageJ application. The mean size value was around 11 nm. The difference between this result and the one obtained from DLS comes from the fact that DLS measures a hydrodynamic diameter of nanoparticles, which is larger than the nanoparticles’ actual diameter. 

It has been observed that Ni nanoparticles redissolve within about 60 min, if the samples are in contact with air. This reaction can be written as follows:

(3) $$Ni^{0}+\frac{1}{2}O_{2}=NiO$$

followed by:

(4) $$NiO+2H^{+}=Ni^{2+}+H_{2}O$$

Ag@NiNPs particles remain stable for over 1 month. During this time, their catalytic properties also remain unchanged. A test was performed using dimethylglyoxime for the presence of Ni^2+^ ions. The test result was negative, which means that there was no secondary dissolution of Ni from Ag@NiNPs. This suggests that the outer layer of Ag was tight, or the resulting AgNi alloy was very resistant to corrosion. However, this requires further research.

#### 3.3.5. XPS Analysis

In order to study the chemical properties of the obtained colloids, the XPS method was used. For this purpose, all colloids needed to be evaporated (in a protective gas atmosphere) and then transported to the measuring instrument. In our opinion, the time associated with the transport of samples between laboratories had little impact on the composition of the samples obtained. However, it cannot be ruled out, which is why we emphasize this fact. The elemental composition of the samples is given in Table 1.

**Figure 8:**
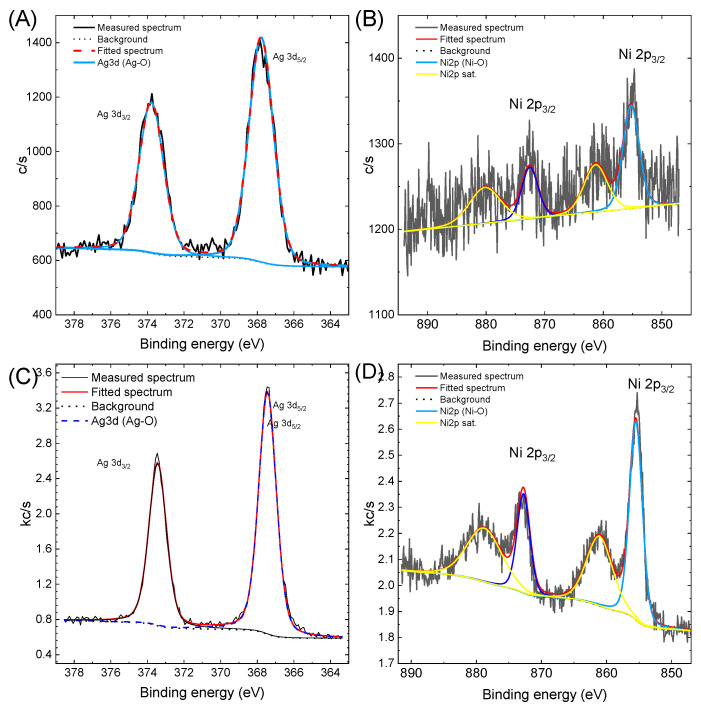
XPS analysis of (**A**) AgNPs, (**B**) NiNPs, (**C**) Ag electronic state in Ag@NiNPs, and (**D**) Ni electronic state in Ag@NiNPs.

Next, a detailed analysis of the electronic states of Ni, Ag, and Ni and Ag in Ag@NiNPs was performed. The obtained results, as well as the analysis, are shown in Figure 8. 

The Ag3d line for the Ag and AgNi samples revealed the presence of Ag in the oxide state (367.4 eV, DS = 6.0 eV) [44], as well as in the metallic state (367.85 eV) [45]. The presence of the metallic state was also confirmed by the presence of surface plasmon resonance in the UV–Vis spectrum (see Figure 1). The Ni2p line was fitted by one doublet and two satellite lines. It revealed the presence of Ni in the oxide state (855.4 eV, DS = 17.3 eV) in both the Ni and AgNi samples [46]. This result is seemingly inconsistent with the XRD results for NiNPs. XRD showed the presence of both nickel oxide and nickel metal. It is worth noting that the XPS method mainly analyzes the surface of the samples. According to Najib A., XPS is a surface-sensitive technique and has an analytical depth of 1–5 nm; consequently, for the analysis of metal-based coatings, it is necessary to remove any surface oxide prior to analyses [47]. In the studied case, the external layer is silver; therefore, the metallic nickel core may not be analyzed by XPS, since the diameter of the Ni-base particles is ca. 10 nm. Thus, only the alloy layer is analyzed by XPS, while XRD gives information from the entire particle, both the core and the shell (see Figure 4).

### 3.4. Catalytic Tests

Noble metals are well-known for their catalytic activity in the chemical reduction/oxidation of dyes. This study evaluated the catalytic activity of AgNPs and Ag@NiNPs using methylene blue (MB) as a model dye pollutant in an aqueous medium.

**Figure 9:**
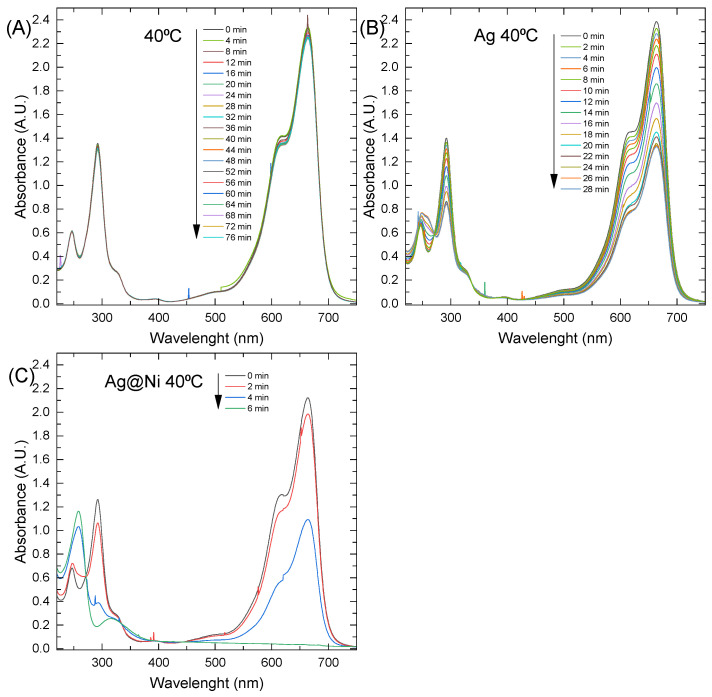
UV–Vis spectra for the reduction of methylene blue by NaBH4 with (**A**) no catalyst, (**B**) AgNPs, and (**C**) AgNiNPs.

As shown in Figure 9A, NaBH_4_ alone could not reduce methylene blue. More precisely, the kinetics of this reaction were very slow, even though the redox potential difference for MB and LMB was small at 0.01 V. A catalyst is needed to lower the reaction activation energy barrier, which would work as an electron mediator between MB and NaBH_4_. Metal nanoparticles are great catalysts for this reaction because of their good conductivity and the large number of active sites provided by the high surface area of the very small nanoparticles [48]. The most popular catalysts for this reaction are AgNPs. Silver nanocatalyst is believed to have a redox potential between the acceptor and donor values [49]. 

Systems with AgNPs showed better performance (Figure 9B), but could not achieve a total reduction of MB in any tested temperature and concentration region. This may be caused by the lower catalytic properties resulting from the partial oxidation of the nanoparticles. Um-e-Salma Amjad et al. [50] pointed out that the capping agents greatly diminish the catalytic activity of silver nanoparticles. Capping agents form shells around the particles and effectively allow for good dispersion due to the creation of a repulsive force between the particles. However, the catalytic reaction occurs on the surface of the nanoparticles, and the reactants need to overcome the resistance caused by the surfactants in order to be reduced. This additional barrier lowers the efficiency of the catalyst, but increases its stability.

Ag@NiNPs proved to be able to completely reduce methylene blue in a whole range of tested temperatures. As was established, nickel was distributed in the whole nanoparticle shell instead of creating the core of the nanoparticle. As a result, the conjunction of silver and nickel atoms on the surface exhibited a synergistic effect of catalytic properties (see Figure 9C). 

The internal stress caused by the deformation of the crystal resulted in an overall increase in the inner energy of the particles. As was proven in different systems and materials [51,52], the internal energy and elastic strain of the crystal lattice may improve the material’s catalytic properties. This phenomenon reduced the time needed for the complete reduction of MB to as low as 4 min at a temperature of 45 °C. 

**Figure 10:**
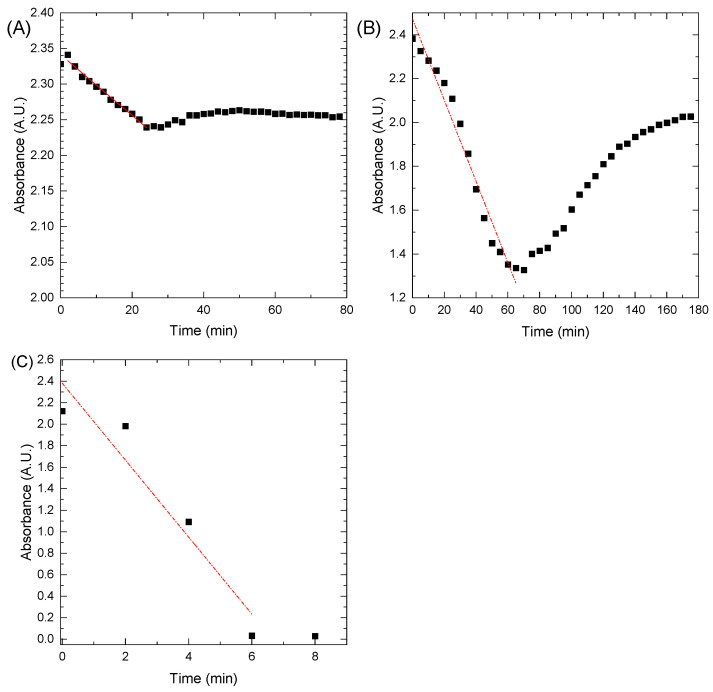
Kinetic curves of the methylene blue reduction process in 40 °C for system (**A**) NaBH_4_, (**B**) AgNPs, and (**C**) AgNiNPs.

The kinetic curves were calculated from the acquired data and used to define the initial reaction rate (labeled as V). Examples of the kinetic curves from the experiments conducted at temperatures equal to 40 °C are presented in Figure 10A–C. As can be seen in Figure 10B, after an hour, the reverse process was observed. The oxidation reaction became dominant, which was shown by increased absorbance on the plot. In the oxidation reaction, oxygen from the air is involved. The increase in the MB concentration is attributed to the depletion of the NaBH_4_ concentration, which showed that even at higher temperatures, this catalyst (AgNP) could not completely reduce methylene blue. It is important to notice the difference in the scales of the plots presented in Figure 10B,C to comprehend the magnitude of the reaction time difference for the studied systems. When AgNiNPs was used, it allowed for the reduction to occur in under 8 min at 40 °C and was the only system that completely reduced MB at a given temperature.

To calculate the activation energy of these two systems, the Arrhenius equation (see Equation (5)) was used:

(5) $$V=Ae^{\left(-\frac{E_{a}}{RT}\right)}$$

where V is the initial reaction rate, A is the pre-exponential factor, R is the gas constant, T equals temperature (K), and E_a_ is the activation energy J/mol.

**Figure 11:**
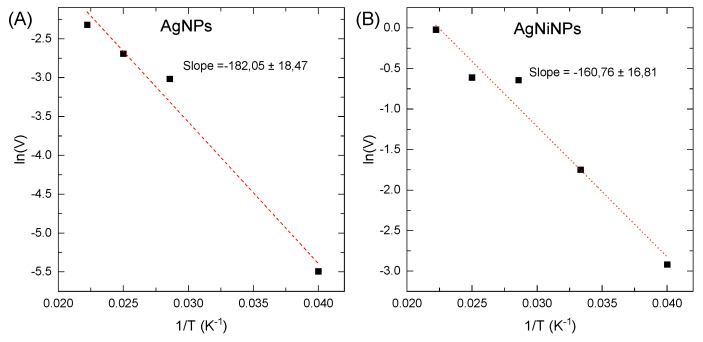
Determination of activation energy using the Arrhenius dependence for (**A**) AgNPs and (**B**) AgNiNPs.

For our calculation, the logarithmic form of the Arrhenius equation was used, in which the activation energy is equal to the negative slope of the curve. Figure 11 shows the curves for (A) AgNPs and (B) AgNiNPs. As shown in Figure 11B, the system with AgNiNPs had a lower activation energy. This reaction rate was higher and processed much faster.

The determined activation energy equaled 1.5 kJ/mol for AgNPs and 1.3 kJ/mol for AgNiNPs. As seen in both cases, the activation energy was low, proving the catalysts’ effectiveness. However, for AgNiNPs, the speed was ten times faster. This was probably related to the much more effective active collisions of MB and NaBH_4_ with the surface of the AgNiNP catalyst. As a result, the alloy nanoparticles showed much better catalytic properties than did pure silver.

S. Mohan and M. V. Devan investigated [53] the photocatalytic activity of AgNi bimetallic nanoparticles on textile dye removal. In their case, the time necessary to remove ca. 100% of the dye was 25 min, whereas in this study, the necessary time was only 8 min. However, it should be noted here that these two processes were significantly different from each other. In the case under study, the reaction rate depended on the initial concentration of NaBH_4_, while in the case of the work by S. Mohan and M. V. Devan, the photochemical processes always depended on the light intensity.

K. Mukesh and D. Sasankacatalytic investigated the reduction of 4-Nitrophenol by NaBH_4_ [43] using AgNi bimetallic nanoparticles. The 4-nitrophenolate ion showed an absorption peak at 400 nm, which remained unchanged in the presence of NaBH_4_, even up to 30 min. This observation is in good agreement with our experimental results. The authors suggested that the reduction process did not occur in the absence of a catalyst due to the presence of a high kinetic barrier between the mutually repelling negative ions of borohydride (BH_4_^−^) and 4-Nitrophenol. This observation is also in good agreement with our kinetic measurements. The low activation energy suggested that a high kinetic barrier existed, and the presence of AgNiNPs was able to effectively accelerate the reduction process. 

## 4. Summary and Conclusions

In this research, we proposed nickel–silver bimetallic nanoparticles as a catalyst for the degradation of organic dyes. For this purpose, methylene blue was chosen as a standard dye and sodium borohydride as the reducing agent. The synergistic effect of the catalytic properties of nickel–silver based nanoparticles was shown. The alloy nanoparticles (diameter 11 nm) allowed for much faster dye reduction by sodium borohydride than single metal silver based nanoparticles.

In conclusion, the creation of alloy nanoparticles took place after the addition of silver to a nickel nanoparticle mixture instead of the formation of the core–shell nanostructure. This was related to the redox potentials of Ni^2+^/Ni^0^ and Ag^+^/Ag^0^. The redox reaction occurred in the case of metallic nickel and silver ions. This led to the reduction of silver and the oxidation of nickel. In the next step, the presence of a reducing agent once again led to the reduction of nickel. Since this was a heterogeneous system, it was energetically favorable to grow existing nickel nanoparticles as an alloy, then create separate silver and nickel nanoparticles mixtures. The possibility of obtaining the alloy nanoparticles from immiscible elements in the macro world allows us to explore various possible catalysts and novel materials. The produced alloy nanoparticles exhibited higher catalytic activity than did the silver nanoparticles, which confirms their possible application as catalysts for other applications.

It is known that MB contains both nitrogen and sulfur in its structure. Since MB dissociates in aqueous solutions, the reaction between MB and NaBH4 was strongly inhibited. When MB was adsorbed on the surface of AgNiNPs, the number of MB degrees of freedom decreased. It is known that highly defective materials show greater adsorption capacity (confirmed by HR-TEM). This explains why the AgNPs exhibited worse catalytic properties than did the AgNiNPs. A full elucidation of this mechanism requires further detailed study.

## Figures and Tables

**Table 1:** XPS elemental analysis. Percentage of total atomic concentration of detected elements.

Sample	% at.					
	C	O	Na	Si	Ni	Ag
Ni	43.9	40.5	10.9	4.0	0.9	-
Ag	31.2	45.2	16.7	6.1	-	0.8
NiAg	48.0	33.6	4.9	6.5	4.3	2.8

## Data Availability

The datasets used and/or analyzed during the current study are available from K. Wojtaszek (kwojtaszek@agh.edu.pl) upon reasonable request.
